# The “Self-Sacrifice” of ImmuneCells in Sepsis

**DOI:** 10.3389/fimmu.2022.833479

**Published:** 2022-04-29

**Authors:** Xiaoyue Wen, Bing Xie, Shiying Yuan, Jiancheng Zhang

**Affiliations:** ^1^ Department of Critical Care Medicine, Union Hospital, Tongji Medical College, Huazhong University of Science and Technology, Wuhan, China; ^2^ Institute of Anesthesia and Critical Care Medicine, Union Hospital, Tongji Medical College, Huazhong University of Science and Technology, Wuhan, China

**Keywords:** sepsis, pyroptosis, NETosis, immune cells, self-sacrifice

## Abstract

Sepsis is a life-threatening organ dysfunction caused by the host’s malfunctioning response to infection. Due to its high mortality rate and medical cost, sepsis remains one of the world’s most intractable diseases. In the early stage of sepsis, the over-activated immune system and a cascade of inflammation are usually accompanied by immunosuppression. The core pathogenesis of sepsis is the maladjustment of the host’s innate and adaptive immune response. Many immune cells are involved in this process, including neutrophils, mononuclear/macrophages and lymphocytes. The immune cells recognize pathogens, devour pathogens and release cytokines to recruit or activate other cells in direct or indirect manner. Pyroptosis, immune cell-extracellular traps formation and autophagy are several novel forms of cell death that are different from apoptosis, which play essential roles in the progress of sepsis. Immune cells can initiate “self-sacrifice” through the above three forms of cell death to protect or kill pathogens. However, the exact roles and mechanisms of the self-sacrifice in the immune cells in sepsis are not fully elucidated. This paper mainly analyzes the self-sacrifice of several representative immune cells in the forms of pyroptosis, immune cell-extracellular traps formation and autophagy to reveal the specific roles they play in the occurrence and progression of sepsis, also to provide inspiration and references for further investigation of the roles and mechanisms of self-sacrifice of immune cells in the sepsis in the future, meanwhile, through this work, we hope to bring inspiration to clinical work.

## 1 Introduction

Sepsis is a systemic inflammatory response syndrome (SIRS) induced by infection, which can cause multiple organ dysfunction in severe cases. Sepsis is accompanied by a release of a variety of inflammatory cytokines secreted from immune cells, including neutrophils, monocytes/macrophages, lymphocytes and microglia (1–3). With the rapid progression of the severity of sepsis and in the critical condition with severe sepsis and septic shock, the average mortality rate of sepsis is 25% to 30%, and even 40%–50% in septic shock (4). Previous studies have demonstrated that the pathological process of sepsis is very complex, which still poses a big challenge in critical care medicine at present (5, 6).

In recent years, the pyroptosis and autophagy, as well as immune cells extracellular traps, including neutrophil extracellular traps (NETs), macrophages extracellular traps (METs), mast cells extracellular traps (MCETs), eosinophils extracellular traps (EETs), basophil extracellular traps (BETs) and dendritic cells extracellular traps (DCETs), are defined as unique forms of programmed cell death, which is different from necrosis and apoptosis (**Table 1**). These are programmed deaths, but none of them are genetically regulated under physiological conditions, they are caused by pathological stimuli or changes in the external environment, then further release of substances by cell death continue to perform essential roles on the body. The death of immune cells to protect or damage the body is undoubtedly similar to a kind of self-sacrificing death of cells, however, they have not been comprehensively evaluated.

**Table 1:** Comparison of different cell death modes.

	Pyroptosis	Apoptosis	Necrosis	NETosis	Autophagy
Initiating	Programmed	Programmed	Accidental	Programmed	Programmed
Inducement	Pathological stimulus	Gene regulation under physiological conditions	Pathological changes or severe damage	Pathological stimulus	Nutrient deficiency or hormone induction
Signaling pathway	Caspase-1/4/5/11	Caspase-3/6/7	Non-caspase	Non-caspase	Non-caspase
Terminal event	Lytic	Non-lytic	Lytic	Lytic	Lytic
TUNEL assay	YES	YES	YES	NO	NO
Plasma membrane pore formation	YES	NO	NO	NO	NO
Organelle	Become deformed	Organelle integrity	Become deformed or swollen	Become deformed	Eaten by autophagosomes
Cellular morphology	Become enlarged and deformed	Shrink	Become enlarged and deformed	Become enlarged and deformed	Produce vacuoles
Effect on tissue	Inflammatory	Non-inflammatory	Inflammatory	Inflammatory	Non-inflammatory
DNA damage	Random degradation	Degraded to 200bp and integer multiples of fragments	Random degradation	No degradation	Random degradation

Researches have confirmed that the pyroptosis is an inflammatory form of programmed cell death dependent on inflammatory caspase-1, caspase-4, caspase-5 and caspase-11, as well as the release of a large number of pro-inflammatory cytokines (3, 7). The pyroptosis mainly relies on inflammasome to activate caspase family and then cleave and activate gasdermin protein, which is transferred to the membrane to form holes and thus leads to cell membrane rupture (8). The role of immune cell pyroptosis in sepsis is by far the most controversial. This self-sacrificing way of death was initially thought to play a protective role in sepsis, but now more and more studies have proved the disadvantages of excessive pyroptosis (8–10).

Immune cells extracellular traps are relatively new as research topics, among which NETs has made significant advances in oncology, autoimmune disease and COVID-19 in recent years (11–13). Neutrophils activate trap and kill pathogens by releasing substances composed of depolymerized chromatin and intracellular granular proteins. Neutrophils would die during the formation of NETs, which is called NETosis (14). With further study, more types of immune cells have been found that they can launch extracellular traps formation with disrupted cell membrane releasing DNA and histone to protect or damage the host tissue in programmed cell death. NETs plays an important role in the clearance of pathogens in sepsis and improves the survival rate of patients with sepsis. Neutrophils release histone, histamine and other inflammatory mediators into the blood after forming NETs, which could damage the tissue and promote the apoptosis of macrophages, aggravating the inflammatory response in sepsis (15). They can activate platelets to start the coagulation process and induce the formation of thrombosis as well, further aggravating the disease (16–18). Therefore, the detailed mechanism of NETs in the pathophysiological process of sepsis deserves further investigation.

The autophagy is a process enveloping bacteria and viruses escaped from the phagosomes or damaged mitochondria into vesicles, fusing with lysosomes to form autophagosomes and degrading their encapsulated contents. The relatively broad term “autophagy” itself has been utilized with rather variable and sometimes misleading connotations. The material in the cytoplasm and lysosomal degradation are acknowledged to be involved in the autophagy (19). In the early stage of sepsis, autophagy is induced in many important organs such as heart, brain, lung, liver and kidney, and it plays a protective role in the body. With the suppression of immune cell function, the body enters a period of continuous immunosuppression, with the development of sepsis, autophagy activity decreased (20). In the progression of sepsis, autophagy of immune cells plays an important role. Many studies have proved that macrophages, lymphocytes and neutrophils play a role in actively removing microorganisms and participating in inflammatory reactions (21–23).

The pyroptosis, autophagy, and immune cells extracellular traps formations can all play significant roles in sepsis in a way that resembles self-sacrifice. Much evidence has shown that pyroptosis, immune cells, extracellular traps formations and autophagy are widely involved in the occurrence and development of infectious diseases, neurological diseases, atherosclerotic diseases, and endocrine disease (14, 19, 24, 25). The immune cells through the self-sacrifice to achieve their goal is very interesting, so far, there are a lot of studies on these three ways of death in sepsis, but whether these three ways play a protective or damaging role in the process of sepsis is controversial at present, and no consistent answers have been obtained in cell experiments or clinical trials. Therefore, an in-depth study of this “self-sacrifice” in sepsis is important and necessary. This review focuses on the roles they play in the occurrence and progression of sepsis. The inspiration and reference could also be provided to us with immune cells’ self-sacrifice in sepsis, which could provide a deeper understanding of the mechanism of sepsis, and new treatment strategies for sepsis.

## 2 The Development of Self-Sacrifice in Immune Cells in Sepsis

### 2.1 The Development of Immune Cell Pyroptosis in Sepsis

The phenomenon of pyroptosis was first discovered by Zychlinsky et al. in 1992. They observed a novel pathogenic mechanism of cell death in *Shigella flexneri*-infected macrophages, they found that this process mainly depended on caspase-1 rather than the traditional form of caspase-3-dependent cell death (26). In 1998, Hubert Hilbi claimed that caspase-1 played an essential role in the specific type of cell death. Pro-interleukin(IL)-1β would be cleaved after the activation of caspase-1, which results in inflammation, and this mode of cell death is distinct from apoptosis. Besides, they proposed that caspase-1 had dual effects of promoting apoptosis and inflammation (27). In 2001, Boise LH and Collins CM first discussed this new type of programmed cell death and used “Pyroptosis” to name the cell death mode dependent on caspase-1 (28, 29). In 2011, Kayagaki et al. highlighted the importance of caspase-11 in pyroptosis and pointed it as a non-classical pathway. Moreover, they demonstrated *in vivo*, as opposed to current reasoning, that caspase-11 rather than caspase-1 might be the basic effector of deleterious inflammatory responses. Their discoveries likewise feature the need to return to the job of caspase-1 versus caspase-11 in various mouse infection models, as so far, all investigations have utilized Casp1/11 twofold knockout mice (30). In 2018, Kambara et al. raised an essential point to explain the pathological mechanisms of sepsis. They reported that gasdermin D (GSDMD), a pro-inflammatory factor, could exert anti-inflammatory effects by promoting neutrophil death, and GSDMD would be a potential therapeutic target in the future (31).

### 2.2 The Development of Immune Cell-Extracellular Traps in Sepsis

In 2004, Volker Brinkmann firstly used IL-8, phosphor myristate (PMA) and lipopolysaccharide (LPS) to stimulate neutrophils. They found that activated neutrophils became flat and formed prominent extracellular structures called NETs, and this process was called NETosis. The fibrous structure of NETs could not only kill bacteria efficiently, but also serve as a physical barrier to prevent the further spread of bacteria. Whereas they observed that the excessive exposure of extracellular histone complexes could cause damage to the immune system, which indicated that NETosis was a double-edged sword to the immune system (32). In 2007, further investigation by Dr. Brinkmann’s group concluded that NETosis was a novel form of cell death pathway. They stressed that NETosis was mainly produced by neutrophils and described the way it killed bacteria effectively postmortem (33). In 2008, for the first time, von Köckritz-Blickwede et al. proposed MECTs as a novel cell death pathway in leukocytes (34). In 2012, Yipp et al. provided evidence that NETosis might lead to tissue damage besides its bactericidal properties (35). In 2015, Boe et al. described extracellular traps in macrophages and called it METosis (36).

### 2.3 The Development of Autophagy-Dependent Immune Cell Death in Sepsis

Ashford TP and Porter KR first proposed the term “autophagy” in 1962 after discovering the phenomenon of “self-eating” in cells (37). It referred to the double layer of membrane shedding from the ribosomal region of the rough endoplasmic reticulum. Autophagosome was formed by the degraded organelles, proteins and other components, which fused with lysosomes to create autophagosome and degraded the encapsulated contents to meet the metabolic needs of the cell itself and the renewal of some organelles (38). In 1997, Matsuura et al. from the Ohsumi Ryoshinori Laboratory discovered that APG1 encodes a new type of serine/threonine protein kinase, and its kinase activity is necessary for autophagy (39). As the molecular mechanism of autophagy has slowly been elucidated, the relationship between autophagy and disease such as oncogenesis, immune system diseases, cardiovascular and neurological diseases has constantly being discovered and understood (40). In 2013, Choi et al. proposed the possible mechanism of autophagy and relevant therapeutic strategies by pharmacological means. Provided for the focal part about AKT-mechanistic target of rapamycin (MTOR) to directing autophagy, they evaluated fasting-induced autophagic reactions and found that they were impeded for hearts for these mice. Moreover, These discoveries showed that the signal defect of impair autophagy caused by LMNA gene mutation is one of the pathogenesis of the disease (41). In 2016, the Nobel Prize in Physiology or Medicine was awarded to Tokyo Institute of Technology researcher Yoshinori Ohsumi due to his discovery of the early identification and characterization mechanisms of the autophagy machinery.

## 3 The Roles and Mechanisms of “Self-Sacrifice” of Immune Cells in Sepsis

### 3.1 The Immune Cells’ Pyroptosis in Sepsis

**Figure 1:**
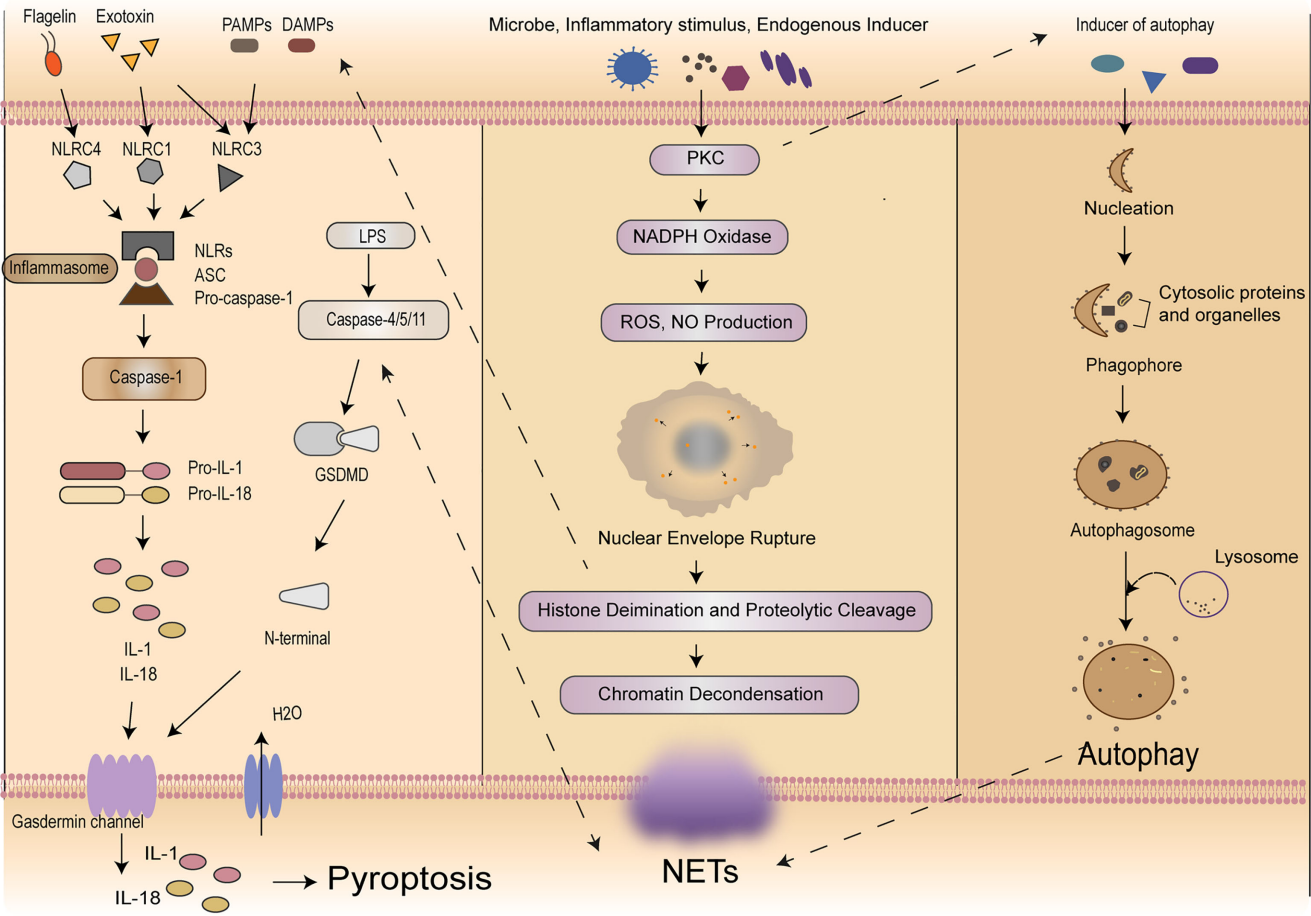
Mechanisms of self-sacrifice in neutrophils. Pyroptosis mainly relies on inflammasome to activate caspase family, caspase family could cleave and activate gasdermin protein, which is transferred to the membrane to form holes and thus leads to cell membrane rupture. NETs components DNA and histones can act as DAMPs molecules to directly initiate or amplify inflammatory responses. NETosis induced by LPS in neutrophils can elicit caspase-11 and GSDMD-dependent histone H3 citrullination. NETosis is a multi-step process involving the destruction of neutrophil nuclear and cytoplasmic granular membranes, chromatin relaxation, chromatin interaction with granular proteins, and chromatin release from the cells. A variety of stimulants, such as PMA, microflora, LPS, eubacteria, activated blood plates, IL-8 and immune complexes, can induce the formation of NETs through the PKC signaling pathway. Autophagy is a process enveloping bacteria and viruses escaped from the phagosomes or damaged mitochondria into vesicles, fusing with lysosomes to form autophagosomes and degrading their encapsulated contents. Neutrophil autophagy could subsequently activate NETs. NETs, neutrophil extracellular traps; DAMPs, damage‐associated molecular patterns; LPS, lipopolysaccharide; GSDMD, gasdermin D; PMA, phosphor myristate; IL, interleukin; PKC, protein kinase C.

According to the cleavage of inflammatory molecules upstream of GSDMD, pyroptosis could be divided into caspase-1-dependent classical pathway and caspase-4/5/11-dependent non-classical pathway (9, 42). In the classical pathway, under the stimulation of pathogens, bacteria and other signals, intracellular Nod-like receptor (NLR) family recognizes these signals and activates casepase-1 by binding with pro-caspase-1 through adaptor protein ASC. On the one hand, activated caspase-1 could cut GSDMD. GSDMD is divided into N-terminal and C-terminal, the N-terminal of GSDMD and phosphatidylinositol on cell membrane are combined to form holes which called “gasdermin channel”, then releasing contents and inducing inflammation (43). On the other hand, activated caspase-1 cleaves pro-IL-1β and pro-IL-18 to transform into IL-1β and IL-18, which is released to extracellular and lead to inflammation as well. In the non-classical pathway, taking LPS as an example, the activation of caspase-4/5/11 does not require the involvement of ASC, LPS could directly enter the cells without the receptors. The lipid A portion of LPS binds to the N-terminal of caspase activation and recruitment domain of caspase-4/5/11 and induces its oligomerization. After caspase-4/5/11 is activated, then the activated caspase-4/5/11 could cleave GSDMD and induce pyroptosis, meanwhile inducing the caspase-1 activation to cleave pro-IL-1β and pro-IL-18, the expression of IL-1β causes a febrile reaction, dilates blood vessels, causes hypotension, promotes immune cells (polymorphonuclear neutrophils, monocytes, macrophages, and dendritic cells) infiltration, and ultimately damages tissues (44). Meanwhile, IL-18 is a cytokine with multiple pro-inflammatory functions, it could activate Th1 cells and produces interferon-γ (IFN-γ). The overproduction of IL-18 may policy the inflammatory response and cause tissue damage (45) resulting in an inflammatory response (43, 46). It is worth noting that pannexin-1and P2X7 downstream of caspase-11 play a significant role for pyroptosis and susceptibility to sepsis induced by the non-classical pathway (47). Then we will focus on exploring how this complex process plays a role in immune cells in sepsis (**Figure 1**).

#### 3.1.1 Neutrophils’ Pyroptosis in Sepsis

Pyroptosis of neutrophils is a pro-inflammatory programmed cell death process performed by the protein GSDMD. From the point of view of protection, the intracellular replicative niche of the bacteria is cleared through pyroptosis of neutrophils, immune factors released during pyroptosis active the effector cells to kill the pathogen (10). In contrast, Mei Yang et al. suggested that neutrophils could secrete IL-1β through caspase-1-dependent classical pathway and then lead to a lower survival rate in mice. Compared to those in controls, the expression of caspase-1, NLRP-1, IL-1β, and IL-18 decreased in mice with AC-YVAD-CMK (caspase-1-inhibitor) treatment, additionally, with AC-YVAD-CMK treatment, they observed that the accumulation of neutrophils and macrophages were suppressed, serum creatinine and blood urea nitrogen level were decreased, and the expression of GSDMD reduced (48). In 2014, Chen et al. confirmed that neutrophils could express multiple Nod Like Receptors (NLRs) and are significant source of IL-1β during acute *Salmonella* infection. In addition, the neutrophil NLRC4 inflammasome drove caspase-1 and IL-1β activation (49). In 2018, Chen et al. clarified that due to the low expression of caspase-1 in neutrophils and the classical inflammasome signal transduction aptamer protein ASC, GSDMD could not be cleaved effectively, resulting in focal death independent of the classical pathway in neutrophils. However, IL-1β could be released during this process, which in turn recruits more neutrophils to accumulate (50).

In caspase-4/5/11-dependent non-classical pathway, it is worth emphasizing that Kumari et al. revealed that the release of caspase-11 in neutrophils was crucial and indispensable for LPS-induced mortality after sepsis, but it does not play primary roles in other cells, such as intestinal epithelial cells (51).

Analogously, during polymicrobial sepsis, Gentile et al. evidenced that the level of inflammatory cytokines and neutrophils’ phagocytosis could increase, the survival rate of wild-type mice and bacterial colonization could decrease after ablation of caspase-1/11, moreover, they emphasize that caspase-1/11 activity, not only could accelerate the magnitude of the inflammatory response, but also suppress protective immunity (52). In contrast, Cheng et al. used the mature neutrophils extracted from mouse bone marrow, and found neutrophils was sensitive to caspase-11-dependent non-classical pathway, in which could effectively cleave the GSDMD in neutrophils, conditional deletion of caspase-11 in lung injury caused by bacterial sepsis could reduce neutrophils accumulation and pyroptosis, indirectly proving that caspase-11-dependent non-classical pathway might be involved in the lung injury after sepsis, moreover, they evidenced that caspase-11 and GSDMD contributed to host defense and the decrease of bacterial load though driving the NETosis (53). Peptidylarginine deiminases (PADs) are a family of calcium-dependent enzymes. In 2020, Yuzi Tian et al. testified that PAD2 protein is increased in sepsis patients and CLP-induced mouse models of sepsis. Through the using of PAD2-specific inhibitors, they demonstrate that it could decrease NETosis and macrophage’casepase-11-dependent pyroptosis, and inhibition of caspase-11 activation could lead to reduce the release of IL-1α and high mobility group box 1 (HMGB1) in the peritoneal cavity, which increases the number of macrophages, significantly reduces bacterial load and inflammation in the blood, and ultimately increases survival and organ function after sepsis (54).

#### 3.1.2 The Lymphocytes’ Pyroptosis in Sepsis

At present, there is no sufficient evidence showing that pyroptosis of lymphocytes can directly aggravate the occurrence of sepsis. Whereas Sarkar et al. used caspase-1 knockout, IL-1 knockout, IL-1/IL-18 double knockout and their respective wild-type mice to analyze the survival rate in 2006, they found that mice with caspase-1 knockout had a higher survival rate and they proposed that caspase-1-dependent pyroptosis might cause delayed apoptosis of B lymphocytes, which further affected the phenotype of macrophages and thus improved the survival rate, of note, this process is not regulated by cytokines, finally they proved that caspase-1 was essential to surviving live *E. coli*-induced septic shock (55). Moreover, studies have shown that Group 2 Innate lymphoid Cells (ILC2) can inhibit the activation of caspase-1 by secreting IL-9, thus inhibiting pyroptosis of vascular endothelial cells, additionally, granzyme released by cytotoxic lymphocytes can trigger pyroptosis in target cells by cleaving GSDMB (56). Kader et al. manifested that in *Ehrlichia*-induced sepsis mice, caspase-11-mediated HMGB1 cytosolic translocation and extracellular secretion are linked to the induction of pyroptosis, the IFNAR signaling involve in this process, which played an essential role in bacterial replication and NKT cells, CD8^+^ cells and neutrophils expanded during this process, resulting the damage of liver in sepsis (57). In a word, the pyroptosis in lymphocytes does have a relationship with sepsis, but the mechanism of their interaction is still worth further exploration.

#### 3.1.3 The Pyroptosis of Monocyte/Macrophage in Sepsis

Sepsis-associated disseminated intravascular coagulation (DIC) could inhibit macrophage pyroptosis through platelet endothelial cell adhesion molecule-1 (PECAM-1), thus restoring vascular barrier integrity (58). PECAM-1 could significantly increase or decrease the expression of caspase-11 through the up-regulation or down-regulation of the expression of Sphingosine-1-phosphate receptors-2 (S1PR2) in macrophages. Many sphingosine-1-phosphate receptors are expressed in macrophages, which could promote caspase-11-dependent pyroptosis of macrophages. Loss of S1PR2 could reduce the pyroptosis of macrophages and improve the prognosis of sepsis caused by *E. coli* infection. They also proved that a RhoA inhibitor significantly reduced caspase-11 activation (59). In 2018, Wang et al. found that the level of the caspase-1, the percentage of caspase-1-induced peripheral blood monocyte pyroptosis, and the level of IL-18 were significantly increased in patients with post-traumatic sepsis compared with healthy subjects, and the percentage of peripheral blood mononuclear cells pyroptosis could predict the development and the occurrence of post-traumatic sepsis (60). In the same year, Chen et al. proved that the activity of bone marrow-derived macrophages in sepsis mice was inhibited after treatment with HMGB1, leading to the increased release of IL-1β and IL-18 through caspase-11-dependent pyroptosis. They demonstrated that HMGB1 interacted with extracellular LPS to intervene caspase-11-dependent pyroptosis in fatal sepsis, which is a physical and specific bond interaction, and in this process RAGE-dependent internalization was a decisive pathway by disrupting the membrane of the acidic lysosome, allowing LPS to enter the cytoplasm. More importantly, they found that HMGB1 and caspase-11 was upregulated 24 hours after the onset of sepsis, providing the basis for targeted therapy for sepsis (61). Unlike caspase-11-dependent pyroptossis in neutrophils, recently studies presented that caspase-11-dependent-pyroptosis in macrophages mainly plays a damaging role in the process of sepsis. Salvamoser et al. showed that the levels of caspase-1 and caspase-11, and the expression of caspase-1 in macrophages were increased in mice after LPS-induced sepsis. Besides, the deficiency of caspase-1 and caspase-11 in mice resulted in increased tolerance to septic shock and reduced mortality in mice (62). Similarly, Kang et al. confirmed that regulating caspase-11-mediated GSDMD cleavage in macrophages improved immune hyperactivation in sepsis, indicating that genetic or pharmacologic suppression of excessive pyroptosis in macrophages could enhance the survival rate of mice in sepsis (63).

Microglia and peripheral macrophages belong to mononuclear macrophages of hematopoietic origin and microglia is the only resident macrophages of the brain parenchyma acting as innate immune effector cells in the central nervous system (64). Microglia activation and neuroinflammation are the main features of neuropathology (65, 66). In 2019, Xu et al. confirmed that caspase-1 inhibitors could suppress the expression of GSDMD and its cleavage from GSDMD-NT, then inhibit the pyroptosis of microglia on day 1 and 7. In addition, it could also reduce the detrimental effects of IL-1β, monocyte chemoattractant protein-1 (MCP-1), and TNF-α in the serum and brain of septic mice and thus alleviate brain injury. Therefore, caspase-1 inhibitors can protect against brain injury caused by sepsis to some extent, mainly by blocking the microglial pyroptosis pathway and reducing the release of pro-inflammatory cytokines (67). Furthermore, Xu et al. further found that that TREM-1 was a key regulator of inflammation. Microglia further increased N-terminal of GSDMD and the formation of GSDMD pores through upregulation of TREM-1, leading to pyroptosis of microglia, then resulting the damage in nerve. TREM-1-induced spleen tyrosine kinase (SYK) mobilization is responsible for microglial pyroptosis through CARD9/NF-κB and NLRP3/caspase-1 pathways both *in vivo* and *in vitro.* It is worth mentioning that such inflammation is not directly equivalent to sepsis, according to the definition of sepsis and their role in sepsis remains to be studied (68). In the same year, Liang Li et al. illustrated that microglial with Cylindromatosis deficiency could exacerbate LPS-induced pyroptosis in septic mice (69). In 2020, a study in a rat *model* of *cardiac* *arrest* found that selective inhibition of NLRP3 and caspase-1 with MCC950 and Ac-YVAD-cmk could significantly prevent microglial pyroptosis (70).

### 3.2 The ICETs Cell Death in Sepsis

NETotic cell death was originally characterized by neutrophils, of note, similar structures have been found in macrophages, eosinophils, basophils, and DC cells by now. This designation has so far been controversial and not widely advocated (71). At present, NETs is mainly through NADPH oxidase (NOX) dependent and NOX independent pathways. A variety of stimulants, such as PMA, microflora, LPS, eubacteria, activated blood plates, IL-8 and immune complexes (IC), could induce the formation of NETs through the NOX-dependent pathway (72–75), protein kinase C (PKC) or rapidly accelerated fibrosarcoma (RAF)-mitogen-activated protein kinase (MEK)-extracellular signal-regulated kinase (ERK) signaling pathway (75, 76). After the activation of neutrophils, NETs can be formed in the NOX-dependent cell death process (77). The NOX-independent pathway occurs without lysis of neutrophils, and ROS generation is not involved in. NETs are transported mainly through nuclear membrane blister and subsequent vesicles. Activated platelets may be the inducer of this pathway. The specific mechanism remains unclear and needs further study. The NETotic cell death is largely unknown, both in terms of naming and mechanism, but its role in sepsis is still being explored.

#### 3.2.1 NETs in Sepsis

NETosis is a multi-step process involving the destruction of neutrophil nuclear and cytoplasmic granular membranes, chromatin relaxation, chromatin interaction with granular proteins, and chromatin release from the cells (14). In 2013, Yipp BG and Kubes P divided NETosis into suicidal NETosis and vital NETosis, and the two categories differ in whether cells are cleaved. Vital NETosis primarily provides extracellular antibacterial action while neutrophils remain mobile and phagocytic (15). Khan et al. added one more point and elucidated that those increasing concentrations of LPS was a critical switch that suicidal NETosis could be turned on (78).

Suicidal NETosis is shown to be able to promote the inflammatory response, induces coagulation disorder, and damages the tissue directly (14, 79). There is growing evidence showing that NETs and their components are cytotoxic and can directly kill endothelial and epithelial cells *in vitro*. Moreover, excessive accumulation of NETs *in vivo* leads to epithelial and endothelial tissue damage (80). Endothelial cell activation is an integral part of sepsis pathogenesis. In the study of Gupta et al. in 2010, activated endothelial cells induced NETs through IL-8 resulting in severe tissue damage (81). What’s more, Villanueva et al. demonstrated that “low-density granulocytes” had a greater capacity to produce NETs. By creating a higher proportion of NETs, these cells might drive disease pathogenesis (82). Xu et al. concluded that Extracellular histones from NETs are cytotoxic *in vitro* and have lethal effect on mice, *in vivo*, histone administration might result in vacuolated endothelium and thrombosis. Besides, After infusing of APC (activated protein C) with E. coli in baboons, they detected that histone in the circulation of baboons decreased and observed it could prevent lethality in mice, implying that APC could cleavage the histone (mainly H3 and H4), and the overactivation of NETs led to extracellular histones accumulation, thus contributed to the death of mice in sepsis (83). Inversely, Schauer et al. in 2014 ascertained that the mice with NETosis-deficient could exacerbate disease that can be decreased by adoptive by aggregated NETs’ transformation. Besides, the hydrolytic protease adhered to NETs interfered with neutrophil recruitment and activation as well as degraded cytokines and chemokines, thereby alleviating local inflammation (84). The above studies show that the specific effects of NETs are being explored and dialectically.

Coagulation disorder is a typical pathological feature of sepsis, and the thrombogenic mechanisms of NETs are varied. First, during the formation of NETs, tissue factors can be produced and released, which act as the trigger for the exogenous clotting pathway, thereby activating the coagulation cascade and simultaneously stimulating platelet activation, leading to platelet aggregation and ultimately thrombosis (85). NETs can then activate endogenous pathways by activating clotting factor XII in the blood, resulting in deep vein thrombosis (86).

Furthermore, pyroptosis and NETosis are not two independent processes in neutrophils. They are mechanistically linked with each other and have mutual effects. One study has shown that NETosis induced by LPS in neutrophils can elicit caspase-11 and GSDMD-dependent histone H3 citrullination. It revealed that neutrophils used an inflammasome and GSDMD-dependent mechanism to activate NETosis as a defense response against cytosolic bacteria. But whether their cooperation is beneficial for removing pathogens still needs more evidence to study. This kind of cooperation is not limited to neutrophils but also in other immune cells.

**Figure 2:**
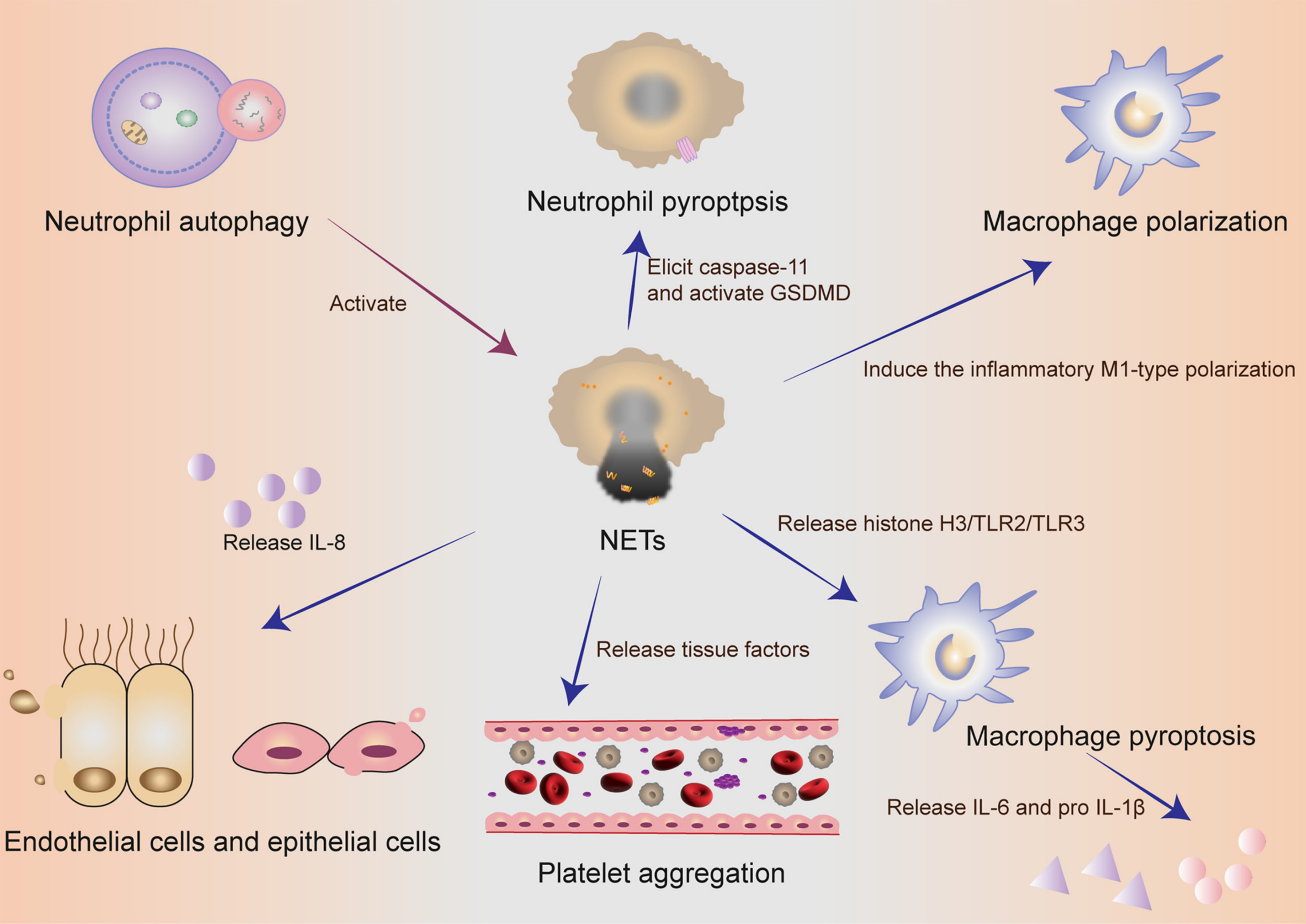
The collaboration of different cells in self-sacrifice. Neutrophil autophagy could subsequently activate NETs. Neutrophils used an inflammasome and GSDMD-dependent mechanism to activate NETosis. NETs could induce the pro-inflammatory M1-type polarization of lung tissue macrophages, thus aggravating lung injury. NETs could promote pyroptosis of macrophages, which could exacerbate the inflammatory response of sepsis. Activated endothelial cells induced NETs through IL-8 resulting in severe tissue damage. NETs and their components are cytotoxic and can directly kill endothelial and epithelial cells. During the formation of NETs, tissue factors can be produced and released, thereby activating the coagulation cascade and simultaneously stimulating platelet activation, leading to platelet aggregation and ultimately thrombosis. NETs, neutrophil extracellular traps; GSDMD, gasdermin D; IL, interleukin.

NETs components DNA and histones can act as damage‐associated molecular patterns (DAMPs) molecules to directly initiate or amplify inflammatory responses (**Figure 2**). Szatmary et al. described the direct damage in three ways, including thrombosis, tissue hypoperfusion and organ damage (80). Tsourouktsoglou et al. found that NETs induced the up-regulation of IL-6 and pro-IL-1β transcription levels in macrophages by TLR2 or TLR4 (87). Similarly, Song et al. in China recently found that NETs could induce the pro-inflammatory M1-type polarization of lung tissue macrophages, thus aggravating lung injury (88). More interestingly, Chen et al. reported that NETs could facilitate macrophage pyroptosis in sepsis through RAGE and dynamin-dependent signaling, in which histone H3 plays an important role (89). At this point, how pyroptosis and NETs cooperate and constrain with each other in neutrophils has been discovered sustainedly in sepsis. Linson Chen further emphasized the connection between NETs and macrophage pyroptosis in sepsis. He stressed out that NETs could promote pyroptosis of macrophages, which could exacerbate the inflammatory response of sepsis (89).

#### 3.2.2 Lymphocytes Extracellular Traps’Nets in Sepsis

Studies have suggested that T lymphocyte depletion is the main feature of immunosuppression in sepsis (90, 91). Immunosuppression of T cells in sepsis could be through cytokines secretion, including the release of IL-10, down-regulation of IL-7, and up-regulation of T cell proliferation by programmed death 1 (PD-1)/PD-1 ligand (PD-L1) (91, 92).

Unfortunately, whether lymphocytes could have extracellular traps’ nets as neutrophils is on doubt and there is no research reported. If lymphocytes also had such structure, we would suppose that the primary function might be as follows (1): a physical barrier and a signal-bearer (2); capturing and killing the bacteria through specific enzyme secretion from extracellular nets (3); killing themselves in programmed cell death, which is caused by excessive activation of lymphocytes, thus giving rise to immunosuppression and exacerbation of sepsis.

#### 3.2.3 Macrophages Extracellular Traps in Sepsis

Extracellular traps exit not only in neutrophils but also in Macrophages. Macrophages extracellular traps (METs) are thought to be composed of cellular DNA, histones and MPO (myeloperoxidase), cellular proteins, which have similar structures with neutrophils. King et al. and Aulik et al. clarified that the increase of ROS might be involved in the pathways (93, 94). Liu et al. proposed a ROS-independent mechanism of METs in the defensive effects against microbes (95). However, whether elastase and myeloperoxidase could contribute to METosis need to be further elucidated (96–98). What is even more interesting is that METs was thought to be capable of killing microorganisms at first defense. However, a recent study noted that the METs induced by a rapid growing mycobacterium release actually provided a scaffold to enhance bacterial growth, and thus facilitated the bacteria’s survival in the disease, intriguingly Sungmo et al. emphasized that this process depended on calcium influx instead of NAPHP oxidase activity, in addition, they found histone, MPO and elastase made up of microbicidal proteins (93). We need to emphasize two points here: firstly, bacterial infection and sepsis are not identical, and further verification is needed to determine whether this mechanism has the same effect in the sepsis models, moreover, PMA-Differentiated THP-1 cells and mycobacterium were used in this trial, which are unique.

#### 3.2.4 Mast Cell-Extracellular Traps in Sepsis

Like NETs, mast cells have appeared to secret their atomic DNA and shape mast cell-extracellular traps (MCETs), which can entangle and eliminate different organisms (34). MCETs mainly consists of histones, tryptase and LL-37 (99). LL-37 an 18-kDa precursor protein, which could defend against bacterial due to its pore-forming activity and induce mast cells to release nucleic acids to kill bacteria (100, 101). MCETs could also recruit neutrophils by storing and releasing TNF-α to further promoting inflammation (102). However, this uncontrolled mast cell degranulation will enhance the nearby immune reaction in this manner, exaggerating and maintaining tissue damage (103–105). Here, we need to point out that the relationship between MCETs and sepsis has not been discussed at present, and the discussion about MCETs mainly focuses on the antibacterial effects (34, 103), and chemical inducers of MCET formation, like listeria monocytogenes and mycobacterium tuberculosis (106–108). Therefore, we expect further studies to point out the close connection between sepsis and MCETs.

#### 3.2.5 Eosinophil and Basophil ExtracellularTraps in Sepsis

Different from NETs, eosinophils release their mitochondrial DNA and granule proteins to form eosinophils extracellular traps (EETs) activated by LPS, IL-5, C5a and eotaxin after response to the physical antibacterial mechanism in the inflammation (109, 110). Notably, its hyperactivation can also be amplified to induce a self-sacrifice attack. Eosinophils can produce some toxins, which are involved in coagulation disorders (111). It is interesting to note that the infiltration of eosinophils to lungs under the infection of *A. fumigates* leads to the degranulation of eosinophils, which provides a protective effect against lethal respiratory virus infections (112). As for basophils, basophils extracellular traps (BETs) has been reported to possess antimicrobial activity by exerting its extracellular traps (104).

#### 3.2.6 Dendritic Cell Extracellular Traps in Sepsis

Dendritic cells (DCs) is specialized antigen presenting cells. DCs play an indirect role mainly by regulating T cells in the progression of sepsis (113). Loures et al. found that plasmacytoid DCs (pDCs) could form pDC extracellular traps (pETs) containing DNA and citrullinated histone H3, which had similar structures with NETs. Infection of *A. fumigatus hyp*hae could stimulate the formation of pETs *via* Dectin-2. It has been shown that pETs acted mainly as an antibacterial agent (114). Besides, a loss of DC autophagy slowed sepsis by increasing lifespan and decreasing IFNα (115).

### 3.3 The Autophagy of Immune Cells in Sepsis

In sepsis, there are multiple targets that could induce autophagy. LPS, as an important pathogenic factor in sepsis, has been shown to induce autophagy through toll-like receptor 4 (TLR4) dependent pathway. TLR4 signaling pathways can be divided into two categories: myeloid differentiation factor 88 (MyD88) dependent pathway and MyD88-independent pathway. LPS activates a downstream P62-dependent aggregation-like inducible structure (ALIS) of selective autophagy through the MyD88-TLR4-dependent pathway (21). In addition, LPS could activate the autophagy by affecting NF-κB in sepsis model (116).

#### 3.3.1 The Autophagy of Neutrophil in Sepsis

At present, there are a majority of studies on the relationship between autophagy and sepsis, and much evidence concludes that autophagy brings a more positive impact on immediate clearance of pathogens (117), neutralization of microbial toxins (118), regulation of cytokine release, and reduction of apoptosis (119). Who has an agreement with the above points is that, Hsieh, C. H has emphasized that in CLP-induced-sepsis mice, the inhibition of autophagy can damage relevant organs, they observed that in the septic hearts the reveal of the autophagosomes could increase dramatically, but few autolysosomes were detected, suggesting that incomplete autophagy might be one of the reasons of tissue damage caused by autophagy (120). On the contrary, a few research results have the opposite conclusion. Autophagy is usually induced in the early phase of sepsis, and its activity decreases in the late stage of sepsis. Studies have also underlined that autophagy was involved in mitochondrial damage caused by sepsis and had toxic effects on the human body, Unuma Kana concluded that LPS stimulated cells to release the exocytosis of autolysosomes, resulting in multi-organ injuries due to its release into the circulation (121).

Autophagy can also promote the formation of NETs (3, 8). In 2017, an analysis of neutrophil autophagy markers in 44 patients with sepsis in South Korea found that neutrophil autophagy increased in patients with sepsis survivors and the autophagy of healthy neutrophil could subsequently activate NETs, which was confirmed *in vivo* in a subsequent study performed in mice, neutrophils isolated from patients who died of sepsis could induced dysregulation of autophagy (72).

#### 3.3.2 The Autophagy of Lymphocyte in Sepsis

Additionally, different from the two-sidedness of pyroptosis and NETosis on immune cells, the primary effect of lymphocyte autophagy activation is to improve the severe inflammatory response of sepsis. In 2017, Oami et al. demonstrated that the lack of autophagy on CD4^+^ T cells will increase the mortality of mice, compared with the control mice, a blockade of autophagy could increase the expression of Bcl-2-like 11, lead to mitochondrial accumulation and accelerate programmed cell death in T cells. And the autophagy of T cells will protect mice from immunosuppression from a control experiment (23). Similarly, in a TLR7-mediated model of autoimmunity, Weindel et al. certified the loss of autophagy in B cells and DCs could lead to excessive tissue inflammation and cytokines related to inflammasome (115). In cecal ligation and puncture (CLP)-induced sepsis model in T-cell-specific autophagy related protein 7 (Atg7)-knockout mice and control mice, the mice with T-cell autophagy deficiency had higher mortality *via* suppressing bacterial clearance in the spleen, they finally proposed that autophagy might inhibit sepsis-induced apoptosis and immunosuppression in T lymphocytes (122). Ge et al. proposed that IL-36β reduced the immunosuppressive activity of CD4^+^ CD25^+^ Tregs by initiating the autophagy, subsequently improving to progress of the host immune reaction in sepsis and reducing the mortality rate in mice. Mechanistic studies uncovered that IL-36β set off autophagy of CD4^+^ CD25^+^ Tregs. These effects were obviously receded under the precondition of the autophagy inhibitor 3-methyladenine or Beclin1 knockdown (123).

#### 3.3.3 The Autophagy of Macrophages in Sepsis

In sepsis, pattern recognition molecules (PRMs), such as LPS from the pathogen, can bind to pattern recognition receptors (PRRs) on the macrophage membrane, and activate downstream inflammatory pathways in macrophages. Activated macrophages express many pro-inflammatory cytokines such as IL-6 and TNF-α, which could promote the inflammatory response of sepsis (128).

In addition to pyroptosis and METs, autophagy also plays a vital role in modulating macrophages in sepsis. Autophagy mainly affects the progress of sepsis by influencing the senescence and phagocytosis of macrophages, regulating the death, polarization and activation of macrophages, and the release of inflammatory cytokines from macrophage (127, 129, 130). The effects of autophagic macrophages on the pathophysiological process of diseases are now debated (131). The excessive activation of macrophages can result in many inflammatory and autoimmune diseases, including sepsis. It has been elucidated that autophagy induced by rapamycin can negatively regulate the abnormal activation of macrophages and reduce the inflammatory response (126).

Autophagy and pyroptosis are not two separate processes in macrophages, they can interact with each other. In a mouse model of *Pseudomonas aeruginosa*-induced sepsis, the deficiency of Atg7 gene, an indispensable regulatory factor in inducing autophagy (132) through phagophore initiation, expansion, transition and fusion (130, 133), could enhance the activity of inflammasomes in macrophages *via* elevating blood levels of IL-1β and IL-18 and increasing macrophage pyroptosis (134). A further study proved that in *Pseudomonas aeruginosa*–induced sepsis, flagellin is an effective activator of the inflammasome, and loss of Atg7 led to increased pyroptosis (135). Consequently, the regulation mechanisms of autophagy in pyroptosis might be a future direction.

## 4 Clinical Research Status

The immune cells die in such a self-sacrificing way in sepsis, which undoubtedly has a huge impact on the procession of sepsis. Based on the above studies, how to control the process of these self-sacrificing immune cells towards the direction of protecting the body is one goal of our clinical struggle. Though the research in this area is extremely limited, there are still several research, which might provide some reference and intelligence for the future clinical research.

Based on the above injury mechanism, treatments for sepsis have been extensively developed now. For instance, nitrosonisoldipine, a photodegradation product of calcium channel inhibitor nisoldipine, can serve as a selective inhibitor of inflammatory caspase to block caspase-1-depenpent classical pyroptosis way and caspase-11-depenpent non-classical way, which ultimately contributes to the improvement of the survival time in LPS- and CLP-induced septic models (124). Similarly, Ethyl pyruvate, is a simple aliphatic ester, which has been proved to inhibit the connection of LPS to caspase-11-depenpent non-classical pyroptosis in macrophages, then positively affects the antibacterial ability of the body in sepsis (136). An Fc-modified HIT-like monoclonal antibody was invented to bind to PF4-NET complexes, further enhance DNase resistance. Treatment with this antibody has the capability of decreasing bacterial dissemination and increasing survival in mouse sepsis models, evidencing a novel NET-targeting approach to improve outcomes in sepsis (125). The same antibody-based strategy was reported in 2020, tetranectin could bind with HGBM-1 to inhibit the recruitment of lactate dehydrogenase and caspase in macrophage, this interaction could further reduce inflammatory damage and play a positive role in lethal sepsis (137).

In 2019, a study showed that Dexmedetomidine could reduce pyroptosis and histone release of astrocytes by reducing NLRP3 and caspase-1 recruitment *in vivo* and *vitro*, which might protect comprehensively nerve cells from damage in sepsis (138). What’ more, there are exploration on treating sepsis through magnesium ions, chemical destruction and of GSDMD, cathelicidin peptide LL-37 (139–141). Although many studies have suggested that caspase and NADPH are potential clinical therapeutic targets in sepsis, unfortunately, they have not been widely practiced and recommended. In 2015, David Nobuhiro Douda proposed a possible therapeutic approach to combat with NETs, which is mediated by ROS and a calcium activated small conductance potassium channel (142).

Although the pathways of pyroptosis, NET_S_ and have attracted a lot of attention from researchers, and their stimulants, pathways, receptors, and effects are increasingly being discovered (143). However, the progress of clinical research and drugs development targeting immune cells is not satisfying. Although targeted drugs have been developed for NETs and pyroptosis like FDA-approved disulfiram and so on (124, 144–148), their roles in immune cells in sepsis remains unclear. In 2020, Stiel et al. collected statistics of complete blood count, C-reactive protein (CRP), IL-6, levels of cell-free DNA (cfDNA), neutrophil elastase (NE) and myeloperoxidase (MPO) in umbilical cord blood to try to predict the early-onset sepsis. Unfortunately, the markers of NETosis they testified could not predict the happening of sepsis (149).

## 5 Summary and Future Research Prospects

Immune cells in previous studies on sepsis mainly revolved around the protective effects of recruiting and activating immune cells on the host and the damage to the body by immunosuppression. In the battle between immune cells and pathogens, if immune cells fail, they will face unprogrammed death, which means necrosis, or apoptosis, but if immune cells could initiate a self-sacrifice programmed death before the failure, for example, T cells and B cells release tissue factors and cytokines to kill bacteria by launching autophagy, and neutrophils and macrophages’ pyroptosis directly or indirectly to improve immune suppression, in this way immune cells will protect the host through sacrificing themselves. Therefore, we separately discussed the function of three different death modes in sepsis immune cells, and this self-sacrificing death mode is intelligent for immune cells. However, not all immune cells can play an active role in protecting the host in this process, as well as neutrophils, macrophages, and microglia, if their pyroptosis and extracellular traps are over-activated, their damage to the organism will be greater than the protective effects in sepsis. (**Tables 2**, **3**) What is worth paying attention to in the future is how to amplify the advantages of self-sacrifice of immune cells, while reducing the disadvantages of self-sacrifice of immune cells. With the continuous discovery of the mechanisms of these self-sacrifice, the corresponding treatments related to sepsis will be the direction of future exploration.

**Table 2:** The pros of various self-sacrifice of immune cells.

Cell types	First author	Method	Results
Neutrophils	Chen et al. (2014) (49)	NETosis induced by caspase-11 and GSDMD	A defense response against cytosolic bacteria↑
	Schauer et al. (2014) (84)	NETsosis	Cytokines and chemokines↓ and local inflammation↑
	Pareja et al. (2013) (117)	Autophagy	Clearance of pathogens↑
	Maurer et al. (2015) (118)	Autophagy	Neutralization of microbial toxins↑
	Liu et al. (2015) (119)	Autophagy	Cytokine release↓ and apoptosis↓
	Hsieh et al. (2011) (120)	Inhibition of autophagy	Damage relevant organs↑
lymphocytes	Zhou et al. (2020) (56)	Inhibiting the activation of caspase-1	Pyroptosis of vascular endothelial cells↓
	Oami et al. (2017) (23)	Lack of autophagy on CD4^+^ T cells	Mortality of mice↑
	Weindel et al. (2017) (115)	Loss of autophagy	Tissue inflammation↑ and cytokines related to inflammasome↑
	Lin et al. (2014) (122)	T-cell autophagy deficiency	Bacterial clearance↓
	Ge et al. (2020) (123)	Initiating the autophagy	Progress of the host immune reaction in sepsis↑ and the mortality rate↓
Monocyte/macrophage	Song et al. (2018) (59)	Reducing the pyroptosis	Prognosis of sepsis↑
	Wang et al. (2018) (60)	Sepsis compared with healthy subjects	Percentage of caspase-1-induced peripheral blood monocyte pyroptosis, and the level of IL-18↑
	Liu et al. (2014) (95)	METs	defensive effects against microbes↑
Eosinophils	Muniz et al. (2018) (112)	EETs	Protective effects against lethal respiratory virus infections↑
basophils	Morshed et al. (2014) (104)	BETs	Antimicrobial activity↑
Dendritic cells (DCs)	Loures et al. (2015) (114)	pETs	Antibacterial activity↑
	Weindel et al. (2017) (115)	Loss of DC autophagy	Lifespan↑ and IFNα↓

GSDMD, gasdermin D; NETs, neutrophil extracellular traps; METs, macrophages extracellular traps; EETs, eosinophils extracellular traps; DC, dendritic cell; BETs, basophil extracellular traps; pETs, pDC extracellular traps; IL, interleukin.

**Table 3:** The cons of various self-sacrifice of immune cells.

Cell types	First author	Method	Results
Neutrophils	Sarkar et al. (2006) (55)	Caspase-1 knockout	Survival rate↑ and expression of IL-1 in neutrophils↓
	Gentile et al. (2015) (52)	Ablation of caspase-1/11	Inflammatory cytokines and neutrophils’ phagocytosis↑, the survival rate of wild-type mice and bacterial colonization↓
	Cheng et al. (2017) (53)	Deletion of caspase-11	Neutrophil accumulation and pyroptosis↓
	Tian et al. (2020) (54)	Inhibiting caspase-11-dependent pyroptosis	Generation of NETs↓ and sepsis severity↓
	Gupta et al. (2010) (81)	Activating endothelial cells	Severe tissue damage↓ through IL-8
	Xu et al. (2009) (83)	Overactivation of NETs	Neutrophil margination, vacuolated endothelium, intra-alveolar hemorrhage and macro- and microvascular thrombosis↑
	Chen et al. (2021) (124)	A selective inhibitor of inflammatory caspase	Survival time↑
	Gollomp et al. (2020) (125)	An antibody of caspase	Bacterial dissemination↓ and survival↑
	Szatmary et al. (2018) (80)	Initiating or amplify inflammatory responses	Thrombosis, tissue hypoperfusion and organ damage↑
	Tsourouktsoglou et al. (2020) (87)	NETs	IL-6↑ and pro-IL-1β transcription levels↑
	Szatmary et al. (2018) (80)	NETs	Epithelial and endothelial tissue damage↑
	Song et al. (2019) (88)	NETs	Pro-inflammatory M1-type polarization of lung tissue macrophages↑
	von Bruhl et al. (2012) (86)	NETs	Deep vein thrombosis↑
	Chen et al. (2018) (89)	NETs	Pyroptosis of macrophages↑ and inflammatory response of sepsis↑
	Yang et al. (2021) (48)	Pyroptosis	Accumulation of neutrophils and macrophages↓, sCR and BUN level↓, the expression of GSDMD↓, the expression of Caspase-1, NLRP-1, IL-1β, and IL-18↓
	Unuma et al. (2015) (121)	Autophagy	Mitochondrial damage caused by sepsis↑ and toxic effects on the human body↑
	Chen et al. (2014) (49)	Pyroptosis	Expression of NLRs↑ and IL-1β↑
Monocyte/macrophage	Luo et al. (2020) (58)	Inhibiting macrophage pyroptosis	vascular barrier integrity↑
	Salvamoser et al. (2019) (62)	Deficiency of caspase1/11	Tolerance to septic shock↑ and the mortality in mice↓
	Kang et al. (2018) (63)	Suppression of excessive pyroptosis	Survival rate of mice↑
	Xu et al. (2019) (67)	Inhibiting the pyroptosis	Brain injury↓
	Wang et al. (2019) (126)	Inhibiting the autophagy	Inflammatory response↓
	Chang et al. (2020) (70)	Selective inhibition of NLRP3	Microglial pyroptosis↓
	Lee et al. (2016) (127)	METs	Bacterial growth and the bacteria's survival in the disease↑
Mast cells	Mollerherm et al. (2016) (103)	MCETs	Tissue damage↑
Eosinophils	Ueki et al. (2013, 2018) (109, 111)	EETs	Coagulation disorders↑

IL, interleukin; TXNIP, thioredoxin interacting protein; sCR, serum creatinine; BUN, Blood Urea Nitrogen; GSDMD, gasdermin D; NLR, NOD-like receptor; METs, macrophages extracellular traps; MCETs, mast cell-extracellular traps; EETs, eosinophils extracellular traps.

What we want to emphasize here is that not all immune cells can produce extracellular traps structures. Eosinophils, neutrophils, basophils, mast cells, macrophages and microglia have been proved to be capable of forming extracellular traps (104, 150). However, some immune cells can only produce extracellular traps in some specific and certain stimulating conditions. Whether the extracellular traps in the immune cells exert positive or negative effects depends on different physiopathology condition, including the different diseases, location of immune cells, immune cell types, and type and intensity of stimulus. But according to the current studies, the extracellular traps of the immune cell are almost a double-edged sword. Whether it is protective or detrimental depends on the pathophysiology of the disease. We propose for the first time here that the structures of histone, enzyme and chromatin DNA secreted by immune cells after specific stimulation are collectively called immune cell-extracellular traps (ICETs). They all contain DNA, histones and granular proteins involved in the innate immune response and die in a programmed way in which their cell membrane is dissolved.

In sepsis, the phenomenon of self-sacrifice of these immune cells is very interesting and notable, which provides us with a novel understanding of the roles and mechanisms of immune cell in the pathogenesis of sepsis. Nevertheless, our discussion is limited in three ways of the self-sacrifice attack in which immune cells die without discussing other ways of self-sacrifice attack. Whether the self-sacrifice attack is elf or devil is still under further study. There have been evidence demonstrating that from the point of view of self-sacrifice, neutrophils do more harm than protection, which means that we could focus on this characteristic in the treatment of sepsis in the future clinical studies. Unfortunately, there is no conclusive evidence proving that the other immune cells do more harm than good through pyroptosis, ICETs or autophagy. The exploration and exploitation of the initiating and regulating pathways, mechanisms of action and potential therapeutic drugs are on the way. On the other hand, how to magnify the protective effects and lower the detrimental effects of “ self-sacrifice “ of immune cells is worth exploring in the future. When to intervene the “ self-sacrifice “ of immune cells is also needed to be clarified. Moreover, the interplay and relationship between pyroptosis, ICETs and autophagy are also an important challenge for future research. Finally, it is worth mentioning that it is regrettable that we have not seen an attempt based entirely on this principle in clinical practice, but it is gratifying to see that there is an upper pathway in the use of a few drugs, and we believe that this path will be gradually explored.

In addition to the immune cells, pyroptosis, extracellular traps or autophagy has also been observed in tissue cells, including cardiomyocytes and endothelial cells. Meanwhile, our review does not cover all immune cells, only several relatively important immune cells are chosen as represents to be discussed.

## Author Contributions

XW and JZ performed the study design, data collection, data analysis, data interpretation, and writing. XW and BX performed the data collection and data analysis. SY and JZ performed the study design, data interpretation, and writing. All authors contributed to the article and approved the submitted version.

## Funding

This work was supported by the National Natural Science Foundation of China (Grant No. 82071480 [to JZ]).

## Conflict of Interest

The authors declare that the research was conducted in the absence of any commercial or financial relationships that could be construed as a potential conflict of interest.

## Publisher’s Note

All claims expressed in this article are solely those of the authors and do not necessarily represent those of their affiliated organizations, or those of the publisher, the editors and the reviewers. Any product that may be evaluated in this article, or claim that may be made by its manufacturer, is not guaranteed or endorsed by the publisher.
